# Finding Cactus Roots in Polynomial Time

**DOI:** 10.1007/s00224-017-9825-2

**Published:** 2017-11-21

**Authors:** Petr A. Golovach, Dieter Kratsch, Daniël Paulusma, Anthony Stewart

**Affiliations:** 1 Department of Informatics, University of Bergen, PB 7803, 5020 Bergen, Norway; 2 Laboratoire d’Informatique Théorique et Appliquée, Université de Lorraine, 57045 Metz Cedex 01, France; 3 Department of Computer Science, Durham University, Durham, DH1 3LE UK

**Keywords:** Square root, Cactus, Treewidth, Clique number

## Abstract

A graph *H* is a square root of a graph *G*, or equivalently, *G* is the square of *H*, if *G* can be obtained from *H* by adding an edge between any two vertices in *H* that are of distance 2. The Square Root problem is that of deciding whether a given graph admits a square root. The problem of testing whether a graph admits a square root which belongs to some specified graph class $\mathcal {H}$ is called the $\mathcal {H}$-Square Root problem. By showing boundedness of treewidth we prove that Square Root is polynomial-time solvable on some classes of graphs with small clique number and that $\mathcal {H}$-Square Root is polynomial-time solvable when $\mathcal {H}$ is the class of cactuses.

## Introduction

Squares and square roots are well-known concepts in graph theory that have been studied first from a structural perspective [27, 30] and later also from an algorithmic perspective (as we will discuss). The *square* *G* = *H* ^2^ of a graph *H* = (*V*_*H*_, *E*_*H*_) is the graph with vertex set *V*_*G*_ = *V*_*H*_, such that any two distinct vertices *u*, *v* ∈ *V*_*H*_are adjacent in *G* if and only if *u* and *v* are of distance at most 2 in *H*. A graph *H* is a *square root* of *G* if *G* = *H* ^2^. It is a straightforward exercise to check that there exist graphs with no square root, graphs with a unique square root as well as graphs with many square roots.

In this paper we consider square roots from an algorithmic point of view. The corresponding recognition problem, which asks whether a given graph admits a square root, is called the Square Root problem. Our research is motivated by the result of Motwani and Sudan [26] who proved in 1994 that Square Root is NP-complete. Afterwards, Square Root was shown to be polynomial-time solvable for various graph classes, such as planar graphs [23], or more generally, any non-trivial minor-closed graph class [28]; block graphs [21]; line graphs [24]; trivially perfect graphs [25]; threshold graphs [25]; graphs of maximum degree 6 [4] and graphs of maximum average degree smaller than $\frac {46}{11}$ [14]. It was also shown that Square Root is NP-complete for chordal graphs [18]. We refer to [4, 5, 14] for a number of parameterized complexity results on Square Root.

The computational hardness of Square Root also led to a variant, which asks whether a given graph has a square root that belongs to some specified graph class $\mathcal {H}$. We denote this problem by $\mathcal {H}$-Square Root. The $\mathcal {H}$-Square Root problem is known to be polynomial-time solvable if $\mathcal {H}$ is the class of trees [23], proper interval graphs [18], bipartite graphs [17], block graphs [21], strongly chordal split graphs [22], graphs with girth at least *g* for any fixed *g* ≥ 6 [11], ptolemaic graphs [19], 3-sun-free split graphs [19] (see [20] for an extension of the latter result to other subclasses of split graphs). In contrast, NP-completeness of this problem has been shown if $\mathcal {H}$ is the class of split graphs [18], chordal graphs [18], graphs of girth at least 4 [11] or graphs of girth at least 5 [10].

It follows from a result of Harary, Karp and Tutte [16] that every square root *H* of a planar square has maximum degree at most 3 and only contains blocks that are of size 4 or isomorphic to an even cycle. It follows from this that such graphs *H* have bounded treewidth. By “blowing up” each bag of a tree decomposition by adding all neighbours of every vertex *u* to every bag that contains *u*, we get a tree decomposition of *H* ^2^. Hence, planar squares have bounded treewidth. As such we may use Courcelle’s Theorem [6] to obtain an alternative (but comparable) proof for the polynomial-time result of Lin and Skiena [23] for Square Root restricted to planar graphs. We note that the polynomial-time algorithms for solving Square Root for graphs of maximum degree at most 6 [4] and graphs of maximum average degree less than $\frac {46}{11}$ [14] are also based on showing that the graphs which permit square roots also have bounded treewidth. Nestoridis and Thilikos [28] proved their result for minor-closed graph classes by showing boundedness of carving width. It is also possible, by using the graph minor decomposition of Robertson and Seymour [29], to show that squares of graphs from minor-closed classes have in fact bounded treewidth. Hence, it is a natural question to ask whether the technique of showing boundedness of treewidth can be used for recognizing some other squares as well. This is the main focus of our paper.

### Our Results

Our results are twofold. First, in Section 3 we focus on the $\mathcal {H}$-Square Root problem for a specific class $\mathcal {H}$ of graphs, namely we let $\mathcal {H}$ be the class of cactuses. A connected graph is a *cactus* if every edge of it is contained in at most one cycle. We give an *O*(*n* ^4^)-time algorithm for solving $\mathcal {H}$-Square Root on *n*-vertex graphs where $\mathcal {H}$ is the class of cactuses. Our research is motivated by the nontrivial question as to whether $\mathcal {H}$-Square Root is polynomial-time solvable if $\mathcal {H}$ is the class of planar graphs, that is, whether squares of planar graphs can be recognized in polynomial time. The known result that squares of trees, which form a subclass of the class of cactuses, can be recognized in polynomial time [23] can be seen as a first step towards solving this problem. As every cactus is planar, our result can be seen as a second step towards solving it. As a side note, cactuses are not a subclass of any of the other aforementioned classes of which the squares can be recognized in polynomial time.

**Fig. 1:**
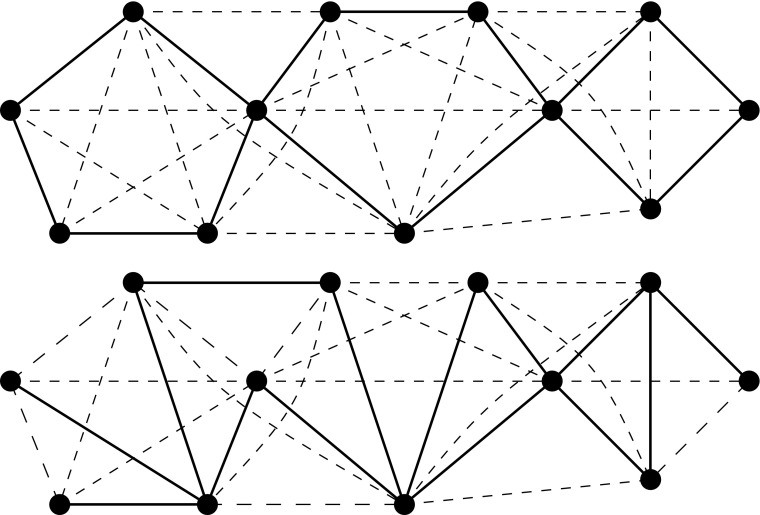
A graph with non-isomorphic square cactus roots. The edges of the cactus roots are shown by solid lines, whereas the other edges are shown by dashed lines

If a graph has a square root that is a cactus, we say that *G* has a *cactus root*. We observe that a general technique applied in several papers [1, 10, 11, 23] is not applicable for finding cactus roots. In these papers the aim is to find some type of sparse square root and it can be shown that such a square root (if it exists) is unique or unique up to isomorphism. This uniqueness can be exploited and as such is very helpful for finding the square root. However, this is not the case for cactus roots; Fig. 1 shows a graph that has two non-isomorphic cactus roots. Instead, we prove our result by showing boundedness of treewidth.

We first analyze, in Section 3.1, the structure of squares of cactuses. This helps us to recognize vertices of the input graph *G* that are cut-vertices in any cactus root (if such a square root exists) and sets of compulsory and forbidden edges of any cactus root of *G*. In this way we can reduce, in Section 3.2, the graph *G* to a number of smaller instances such that *G* has a cactus root if and only if each of these smaller instances has a cactus root. Showing that each of the smaller instances has bounded treewidth, the aforementioned observation that we can solve the problem in polynomial time on any graph class of bounded treewidth completes the proof.

In Section 4 we focus on the Square Root problem restricted to some classes of graphs that have a small clique number. Our motivation for doing so comes from the observation that Square Root is readily seen to be polynomial-time solvable for graphs with clique number at most 3 (the only square roots a connected graph on *n* vertices with clique number 3 may have are the cycle or path on *n* vertices). Moreover, by identifying such classes of graphs, our results complement existing polynomial-time results for other classes of graphs with a small clique number, such as planar graphs [23] and graphs of maximum degree 6 [4]. We prove that Square Root is polynomial-time solvable for the classes of 3-degenerate graphs and (*K*_*r*_, *P*_*t*_)-free graphs by showing that squares in these two graph classes have bounded treewidth.

In Section 5 we present two tables that incorporate both new and old results and there we also discuss some directions for future work.

## Preliminaries

We consider only finite undirected graphs without loops and multiple edges. We refer to the textbook of Diestel [8] for any undefined graph terminology.

We denote the vertex set of a graph *G* by *V*_*G*_ and the edge set by *E*_*G*_. The subgraph of *G* induced by a subset *U* ⊆ *V*_*G*_ is denoted by *G*[*U*]. The graph *G* − *U* is the graph obtained from *G* after removing the vertices of *U*. If *U* = {*u*}, we also write *G* − *u*. Similarly, we denote the graph obtained from *G* after deleting a set of edges *S*(an edge *e*) by *G* − *S*(*G* − *e* respectively).

Let *G* be a graph. A *connected component* of *G* is a maximal connected subgraph. The *distance* dist_*G*_(*u*, *v*) between a pair of vertices *u* and *v* of *G* is the number of edges of a shortest path between them. The diameter diam(*G*)of *G* is the maximum distance between two vertices of *G*.

The *open neighbourhood* of a vertex *u* ∈ *V*_*G*_ is defined as *N*_*G*_(*u*) = {*v* | *u* *v* ∈ *E*_*G*_}, and its *closed neighbourhood* is defined as *N*_*G*_[*u*] = *N*_*G*_(*u*) ∪{*u*}. Two (adjacent) vertices *u*, *v* are said to be *true twins* if *N*_*G*_[*u*] = *N*_*G*_[*v*]. A vertex *v* is *simplicial* if *N*_*G*_[*v*]is a *clique*, that is, if there is an edge between any two vertices of *N*_*G*_[*v*]. The *clique number* of *G* is the size of a largest clique of *G*.

Let *G* be a graph. The *degree* of a vertex *u* ∈ *V*_*G*_is defined as *d*_*G*_(*u*) = |*N*_*G*_(*u*)|. The *maximum degree* of *G* is Δ(*G*) = max{*d*_*G*_(*v*) | *v* ∈ *V*_*G*_}. A vertex of degree 1 is said to be a *pendant* vertex. If *v* is a pendant vertex, then we say that the unique edge incident to *u* is a *pendant* edge. For a non-negative integer *d*, the graph *G* is said to be *d*-*degenerate* if every subgraph *H* of *G* has a vertex of degree at most *d*.

Let {*H*_1_,…,*H*_*p*_} be a family of graphs. Then a graph *G* is called (*H*_1_,…,*H*_*p*_)-*free* if *G* contains no induced subgraph isomorphic to a graph in {*H*_1_,…,*H*_*p*_}. We denote the complete graph and path on *r* vertices by *K*_*r*_ and *P*_*r*_, respectively.

A vertex *u* is a *cut vertex* of a connected graph *G* with at least two vertices if *G* − *u* is disconnected. An inclusion-maximal induced subgraph of *G* that has no cut vertex is called a *block*. Recall that a connected graph *G* is a cactus if each edge of *G* is contained in at most one cycle. This implies the following well-known property.

### **Observation 1**

*Each block of a cactus with at least two vertices is either a* *K*_2_ *(an edge) or a cycle.*

A *tree decomposition* of a graph *G* is a pair (*T*, *X*) where *T* is a tree and *X* = {*X*_*i*_∣*i* ∈ *V*_*T*_} is a collection of subsets (called *bags*) of *V*_*G*_such that the following three conditions hold:

i) $\bigcup _{i \in V_{T}} X_{i} = V_{G}$,
ii) for each edge *x* *y* ∈ *E*_*G*_, *x*, *y* ∈ *X*_*i*_for some *i* ∈ *V*_*T*_, and
iii) for each *x* ∈ *V*_*G*_the set {*i*∣*x* ∈ *X*_*i*_}induces a connected subtree of *T*.

The *width* of a tree decomposition ({*X*_*i*_∣*i* ∈ *V*_*T*_},*T*)is $\max _{i \in V_{T}}\,\{|X_{i}| - 1\}$. The *treewidth* **t** **w**(*G*)of a graph *G* is the minimum width over all tree decompositions of *G*. We will make use of the following result of Bodlaender.

### **Lemma 1** (3)

*For any fixed constant* *k*, *it is possible to decide in linear time whether the treewidth of a graph is at most* *k*.

## Cactus Roots

Recall that a graph *H* is called a cactus root of a graph *G* if *H* is a cactus and a square root of *G*. If $\mathcal {H}$ is the class of cactuses, we may denote the $\mathcal {H}$-Square Root as the following problem:

Cactus Root

**Input:** a graph *G*.
**Question:** is there a cactus *H* with *H* ^2^ = *G*?

We also need to define the following more general variant introduced in [4] for general square roots:

Cactus Root with Labels

**Input:** a graph *G* and sets of edges *R*, *B* ⊆ *E*_*G*_.
**Question:** is there a cactus *H* with *H* ^2^ = *G*, *R* ⊆ *E*_*H*_and *B* ∩ *E*_*H*_ = *∅*?

By choosing *R* = *B* = *∅* we see that Cactus Root is indeed a special case of Cactus Root with Labels.

In Section 3.1 we analyze the structure of squares of cactuses. We use the information obtained in this way for the design of our algorithm, which we describe in Section 3.2.

### A Number of Structural Observations and Lemmas

In this section we state three observations and prove seven lemmas. We will use these results, which are all structural, for the design of our *O*(*n* ^4^) time algorithm for Cactus Root presented in Section 3.2.

The first observation is known and easily follows from the definition of the treewidth.

#### **Observation 2**

*For a cactus* *G*, **t** **w**(*G*) ≤ 2.

The second observation gives an upper bound for the treewidth of the square of a graph; it follows from the well-known fact that we can transform every tree decomposition (*T*, *X*) of a graph *G* into a tree decomposition of *G* ^2^ by adding, to each bag *X*_*i*_of *T*, all the neighbours of every vertex from *X*_*i*_.

#### **Observation 3**

*For a graph* *G*, **t** **w**(*G* ^2^) ≤ (**t** **w**(*G*) + 1)(Δ(*G*) + 1) − 1.

Let *H* be a square root of a graph *G*. We say that *H* is a *minimal* square root of *G* if *H* ^2^ = *G* but any proper subgraph of *H* is not a square root of *G*. Note that the two cactus roots displayed in Fig. 1 are both minimal. Since any connected subgraph of a cactus is a cactus, we can make the following observation.

#### **Observation 4**

*If a graph* *G* *has a cactus root, then* *G* *has a minimal cactus root.*

A block of a graph *G* is called a *leaf block* if it contains at most one cut vertex of *G*. This leads to our first lemma.

#### **Lemma 2**

*If a cactus* *H* *is a minimal square root of a graph* *G*, *then* *H* *has no leaf block that is a triangle.*

#### *Proof*

Suppose that a cactus *H* is a minimal square root of *G* such that a triangle with vertices *x*, *y*, *z* is a leaf block of *H*. As a leaf block contains at most one cut vertex of *H* by definition, we may assume that *y* and *z* are not cut vertices of *H*. Let *H* ^′^ = *H* − *y* *z*. It is straightforward to verify that *H* ^′2^ = *G*, contradicting the minimality of *H*. □

Suppose that *u* and *v* are pendant vertices of a square root *H* of *G* and that *u* and *v* are adjacent to the same vertex of *H* −{*u*, *v*}. Then, in *G*, *u* and *v* are simplicial vertices and true twins. We use this observation in the proof of the following lemma.

#### **Lemma 3**

*Let* *H* *be a minimal cactus root of a graph* *G*. *If* *G* *contains at least six simplicial vertices that are pairwise true* *twins, then at least one of these vertices is a pendant vertex* *of* *H*.

#### *Proof*

Let *H* be a minimal cactus root of a graph *G* that contains a set *X* of six simplicial vertices that are pairwise true twins. The vertices of *X* cannot all belong to the same block of *H*, because such a block would be a cycle with at least six vertices (by Observation 1) and any two vertices of this block could not be true twins of *G*. Hence, there is a cut vertex *u* of *H* such that there exist two vertices *x*, *y* ∈ *X* that are in distinct connected components of *H* − *u*. Let *H* ^′^be a connected component of *H* − *u* that contains *x*. If *x* is not a pendant vertex of *H* then, by the minimality of *H* and Lemma 2, there exists a vertex *z* ∈ *V*_*H*_^′^ that is adjacent to *x* and that is at distance 2 from *u* in *H*. Then, as every path from *y* to *z* in *H* contains *u*, we find that *y* *z*∉*E*_*G*_. This is a contradiction since *x* and *y* are true twins of *G* and *x* *z* ∈ *E*_*G*_. We conclude that *x* is a pendant vertex of *H*. □

The following definition plays a crucial role in our paper.

#### **Definition 1**

Let *u* be a cut vertex of a connected graph *H*. We say that

(i) *u* is *important* if *H* − *u* has three vertices that belong to three distinct connected components of *H* − *u* and that are each at distance at least 2 from *u* in *H*;
(ii) *u* is *essential* if *H* − *u* has two vertices that belong to two distinct connected components of *H* − *u* and that are both at distance at least 2 from *u* in *H*.

Definition 1(i) immediately implies the following lemma.

#### **Lemma 4**

*If* *u* *is an important cut vertex of a cactus root* *H* *of a graph* *G*, *then there are three vertices* *x*, *y*, *z* ∈ *N*_*G*_(*u*) *such that* *x*, *y* *and* *z* *are at distance at least* *3 from each other in* *G* − *u*.

Although we have no implication in the opposite direction, we can show the following (which explains why we need the second and weaker part of Definition 1).

#### **Lemma 5**

*Let* *G* *be a graph with a cactus root* *H*. *If* *u* ∈ *V*_*G*_ *has three neighbours* *x*, *y* *and* *z* *in* *G* *that are at distance at least* *3 from each other in* *G* − *u*, *then* *u* *is an essential cut vertex of* *H*. *Moreover, at least two vertices of* {*x*, *y*, *z*} *belong to distinct connected components of* *H* − *u*.

#### *Proof*

Assume that *G* has a cactus root *H*. Let *u* ∈ *V*_*G*_ be such that *u* has three neighbours *x*, *y* and *z* in *G* that are at distance at least 3 from each other in *G* − *u*. Notice that because *x*, *y* and *z* are at distance at least 3 from each other in *G* − *u*, these vertices are all at distance 2 from *u* in *H*.

For contradiction, assume that *u* is not a cut vertex of *H*. Then *u* has at most two adjacent vertices in *H*, since *H* is a cactus (see Observation 1). Then at least two vertices of {*x*, *y*, *z*}are adjacent to the same vertex of *H*(which is one of the two neighbours of *u*) implying that these two vertices of {*x*, *y*, *z*}are adjacent in *G* and thus in *G* − *u*; a contradiction. Hence *u* is a cut vertex of *H*.

Now suppose that *x*, *y* and *z* are all in the same connected component *H* ^′^of *H* − *u*. Since *H* is a cactus, we find, by Observation 1, that *H* ^′^contains at most two vertices that are adjacent to *u* in *H*. Again, we obtain that at least two vertices of {*x*, *y*, *z*}are adjacent to the same vertex of *H*; a contradiction. Hence, at least two vertices of {*x*, *y*, *z*}belong to distinct connected components of *H* − *u*. Since *x*, *y* and *z* are at distance 2 from *u* in *H*, this implies that *u* is an essential cut vertex of *H*. □

We now show that we can recognize edges of a cactus root that are incident to an essential cut vertex.

#### **Lemma 6**

*Let* *u* *be an essential cut vertex of a cactus root* *H* *of a graph* *G*. *Then for every* *x* ∈ *N*_*G*_(*u*), *it holds that* *u* *x* ∉*E*_*H*_ *if and only if there exists a vertex* *y* ∈ *N*_*G*_(*u*) *such that* *x* *and* *y* *are at distance at least* *3 in* *G* − *u*.

#### *Proof*

Let *u* be an essential cut vertex of a cactus root *H* of a graph *G*. Let *x* ∈ *N*_*G*_(*u*). First suppose that *u* *x* ∈ *E*_*H*_. Let *y* ∈ *N*_*G*_(*u*). If *u* *y* ∈ *E*_*H*_, then *x* *y* ∈ *E*_*G*_. If *u* *y*∉*E*_*H*_, then there exist a vertex *z* ∈ *V*_*H*_and edges *u* *z*, *z* *y* ∈ *E*_*H*_, as *y* ∈ *N*_*G*_(*u*). As *z* *y* ∈ *E*_*H*_, we find that *z* *y* ∈ *E*_*G*_. As *u* *x*, *u* *z* ∈ *E*_*H*_, we also deduce that *x* *z* ∈ *E*_*G*_. In both cases *x* and *y* are at distance at most 2 in *G* − *u*.

Now suppose that *u* *x*∉*E*_*H*_. Then, as *x* ∈ *N*_*G*_(*u*), we find that *x* is at distance 2 from *u* in *H*. Let *H* ^′^be the connected component of *H* − *u* containing *x*. Since *u* is an essential cut vertex of *H*, *H* − *u* has another connected component *H* ^″^containing a vertex *y* at distance 2 from *u* in *H*. It remains to observe that *y* ∈ *N*_*G*_(*u*)and *x* and *y* are at distance 3 in *G* − *u*. □

The next lemma is used to recognize vertices adjacent to an essential cut vertex that belong to the same block of a minimal cactus root.

#### **Lemma 7**

*Let* *H* *be a minimal cactus root of a graph* *G*. *For any* *u* ∈ *V*_*H*_, *two distinct vertices* *x*, *y* ∈ *N*_*H*_(*u*) *are in the same block of* *H* *if and only if* *x* *and* *y* *are in the same connected component of* *G* ^′^ = *G* − *E*_*G*_[*N*_*H*_(*u*)] − *u*.

#### *Proof*

Let *x*, *y* ∈ *N*_*H*_(*u*). First suppose that *x* and *y* are in distinct blocks of *H*. Then *x* and *y* are readily seen to be in distinct connected components of *G* ^′^. Now suppose that *x* and *y* are in the same block *C* of *H*. If *x* *y* ∈ *E*_*G*_then *x* and *y* are in the same connected component of *G* ^′^. Suppose *x* *y*∉*E*_*G*_. Then *C* is a cycle by Observation 1. If *C* is not a triangle, then *C* has a unique (*x*, *y*)-path in *H*(avoiding *u*) of length at least 2. This path is an (*x*, *y*)-path in *G* ^′^as well. Hence *x* and *y* are in the same connected component of *G* ^′^. Suppose that *C* is a triangle.Then *x* *y* ∈ *E*_*H*_. As *H* is a minimal cactus root, *x* or *y* has at least one neighbour *z*≠*u* in *H* due to Lemma 2. Assume without loss of generality that *z* is a neighbour of *x*. Then the edges *x* *y*, *x* *z* ∈ *E*_*H*_imply that *z* *y* ∈ *E*_*G*_. We establish that *x* *z* *y* is an (*x*, *y*)-path in *G* ^′^, that is, also in this case *x* and *y* are in the same connected component of *G* ^′^. □

Finally we show how to determine which neighbours in *G* of an essential cut vertex *u* of a cactus root *H* are in the same connected component of *H* − *u*.

#### **Lemma 8**

*Let* *H* *be a minimal cactus root of a graph* *G*. *For any* *u* ∈ *V*_*H*_ *and* *x* ∈ *N*_*H*_(*u*), *a vertex* *y* ∈ *N*_*G*_(*u*) *is in the same connected component of* *H* − *u* *as* *x* *if and only if either* *u* *y* ∈ *E*_*H*_ *and* *y* *in the same block of* *H* *as* *x*, *or* *u* *y* ∉ *E*_*H*_ *and there is a vertex* *z* ∈ *N*_*H*_(*u*), *such that* *z* *is in the same block of* *H* *as* *x* *and* *y* *z* ∈ *E*_*G*_.

#### *Proof*

Let *y* ∈ *N*_*G*_(*u*). First suppose *y* is in the same connected component of *H* − *u* as *x*. If *u* *y* ∈ *E*_*H*_, then *y* is in the same block of *H* as *x*. Suppose *u* *y*∉*E*_*H*_. As *u* *y* ∈ *E*_*G*_, there is a vertex *z* ∈ *N*_*H*_(*u*)such that *z* *y* ∈ *E*_*H*_. Then *z* is in the same block of *H* as *x*, as *x* and *y* are in the same connected component of *H* − *u*.

To prove the reverse implication, if *u* *y* ∈ *E*_*H*_and *x*, *y* are in the same block of *H*, then *x* and *y* are in the same connected component of *H* − *u*. Suppose that *u* *y*∉*E*_*H*_and there is a vertex *z* ∈ *N*_*H*_(*u*)such that *z* is in the same block of *H* as *x* and *y* *z* ∈ *E*_*G*_. If *y* *z* ∈ *E*_*H*_, then *y* and *z* are in the same connected component of *H* − *u*. If *y* *z*∉*E*_*H*_, then there is a *v* ∈ *V*_*G*_such that *y* *v*, *v* *z* ∈ *E*_*H*_. Since *u* *y*∉*E*_*H*_, we obtain *v*≠*u*. Therefore, *y* and *z* are in the same connected component of *H* − *u*. Because *y* and *z* are in the same connected component of *H* − *u* and *x*, *y* are in the same block of *H*, we obtain that *x*, *y* are in the same connected component of *H* − *u*. □

### The Algorithm

In this section we use the structural results from the previous section to obtain a polynomial-time algorithm for Cactus Root. The main idea is to reduce a given instance of Cactus Root to a set of smaller instances of Cactus Root with Labels, each having bounded treewidth. We therefore need the following lemma which show, together with Lemma 1 and Observations 2 and 3, that we are done if we manage to achieve this goal.

#### **Lemma 9**

Cactus Root with Labels *can be solved in time* *f*(*t*) ⋅ *n* *for* *n* *-vertex* *graphs of treewidth at most* *t*.

#### *Proof*

It is not difficult to construct a dynamic programming algorithm for the problem (for details see [4] in which such an algorithm is sketched for the general Square Root problem). For simplicity we give a non-constructive proof based on Courcelle’s theorem [6]. By this theorem, it suffices to show that the existence of a cactus root can be expressed in monadic second-order logic.

Let (*G*, *R*, *B*) be an instance of Cactus Root with Labels. We observe that the existence of a cactus *H* such that *G* = *H* ^2^, *R* ⊆ *E*_*H*_ and *B* ∩ *E*_*H*_ = *∅* is equivalent to the existence of a subset *X* ⊆ *E*_*G*_such that the following four properties hold:

(i) *R* ⊆ *X* and *B* ∩ *X* = *∅*;
(ii) for every *u* *v* ∈ *E*_*G*_, *u* *v* ∈ *X* or there exists a vertex *w* such that *u* *w*, *w* *v* ∈ *X*;
(iii) for every two distinct edges *u* *w*, *v* *w* ∈ *X*, *u* *v* ∈ *E*_*G*_;
(iv) for every *u* *v* ∈ *X* and for every two (*u*, *v*)-paths *P*_1_and *P*_2_in *G* such that *E*_*P*__1_, *E*_*P*__2_ ⊆ *X* ∖{*u* *v*}, it holds that *P*_1_ = *P*_2_.

Each of these properties can be expressed in monadic second-order logic. In particular, with respect to property (iv), expressing that a subgraph *P* of *G* is a (*u*, *v*)-path in *G* can be done in monadic second-order logic in a standard way (see, for example, [7]). Hence the lemma follows. □

Now we are ready to prove the main result.

#### **Theorem 1**

Cactus Root *can be solved in time* *O*(*n* ^4^) *for* *n* *-vertex* *graphs.*

#### *Proof*

**Fig. 2:**
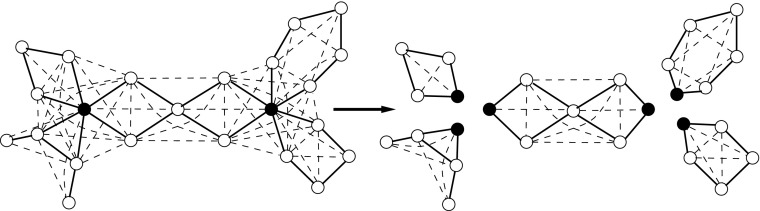
Splitting of a graph; the vertices of *U* are black, the edges of a square root are shown by solid lines and the other edges are shown by dashed lines

We first give an overview of our algorithm. As we can consider each connected component separately, we may assume without loss of generality that the input graph *G* is connected. First, we use Lemma 3 to recognize sets of pendant vertices in a (potential) cactus root adjacent to the same vertex that have size at least 7. For each of these sets, we show that it is safe to delete some vertices without changing the answer for the considered instance. After performing this step, we obtain a graph *G* ^′^such that in any cactus root of *G* ^′^each vertex is adjacent to at most six pendants. Further, we use Lemmas 4 and 5 to construct a set *U* of essential cut vertices in a (potential) cactus root such that *U* contains all important cut vertices. Next, we apply Lemma 6 to recognize which edges incident to the vertices of *U* are in any cactus root and which edges are not included in any cactus root. We label them red and blue respectively and obtain an instance of Cactus Root with Labels. Now we can use Lemmas 7 and 8 to determine for each *u* ∈ *U*, the partition of the set of vertices of *G* − *u* into the sets of vertices of the connected components of *H* − *u*, where *H* is a cactus root of *G* ^′^. This allows us to split *G* ^′^via the vertices of *U* as shown in Fig. 2. Due to the presence of labelled edges incident to the vertices of *U*, we obtain an equivalent instance. Finally, we observe that the obtained graph has bounded treewidth using Observations 2 and 3, so we can use Lemmas 1 and 9 to solve the problem, as we pointed out already. □

Now we formally explain the details of our algorithm. Let *G* be a connected graph. First, we preprocess *G* using Lemma 3 to reduce the number of pendant vertices adjacent to the same vertex in a (potential) cactus root of *G*. To do so, we exhaustively apply the following rule.

#### **Pendants Reduction**

If *G* has a set *X* of simplicial true twins of size at least 7, then delete an arbitrary *u* ∈ *X* from *G*.

The following claim shows that this rule is safe.

#### **Claim A**

*If* *G* ^′^ = *G* − *u* *is obtained from* *G* *by the application of Pendant reduction, then* *G* *has a cactus root if and only if* *G* ^′^ *has a cactus root.*

We prove Claim A as follows. Suppose that *H* is a minimal cactus root of *G*. By Lemma 3, *H* has a pendant vertex *u* ∈ *X*. It is easy to verify that *H* ^′^ = *H* − *u* is a cactus root of *G* ^′^. Assume now that *H* ^′^ is a minimal cactus root of *G* ^′^. By Lemma 3, *H* has a pendant vertex *w* ∈ *X* ∖{*u*}, since the vertices of *X* ∖{*u*} are simplicial true twins of *G* ^′^and |*X* ∖{*u*}|≥ 6. Let *v* be the unique neighbour of *w* in *H* ^′^. We construct *H* from *H* ^′^ by adding *u* and making it adjacent to *v*. It is readily seen that *H* is a cactus root of *G*. This completes the proof of Claim A.

For simplicity, we call the graph obtained by exhaustive application of the pendants rule *G* again. The following property is important for us.

#### **Claim B**

*Every cactus root of* *G* *has at most six pendant vertices adjacent to the same vertex.*

Now we construct an instance of Cactus Root with Labels together with a set *U* of cut vertices of a (potential) cactus root.

#### **Labelling**

Set *U* = *∅*, *R* = *∅* and *B* = *∅*. For each *u* ∈ *V*_*G*_ such that there are three distinct vertices *x*, *y*, *z* ∈ *N*_*G*_(*u*)that are at distance at least 3 from each other in *G* − *u* do the following:

(i) set *U* = *U* ∪{*u*},
(ii) set *B* ^′^ = {*u* *v* ∈ *E*_*G*_∣∃*w* ∈ *N*_*G*_(*u*) s.t. dist_*G*−*u*_(*v*, *w*) ≥ 3},
(iii) set *R* ^′^ = {*u* *v*∣*v* ∈ *N*_*G*_(*u*)}∖ *B* ^′^,
(iv) set *R* = *R* ∪ *R* ^′^and *B* = *B* ∪ *B* ^′^,
(v) if *R* ∩ *B*≠*∅*, then return a no-answer and stop.

Lemmas 4–6 immediately imply the following claim.

#### **Claim C**

*If* *G* *has a cactus root, then Labelling does not stop in Step* *(v), and if* *H* *is a minimal cactus root of* *G*, *then* *R* ⊆ *E*_*H*_ *and* *B* ∩ *E*_*H*_ = *∅*. *Moreover, every vertex* *u* ∈ *U* *is an essential cut vertex of any cactus root of* *G*, *and any important cut vertex* *u* *of any cactus root of* *G* *is contained in* *U*.

For each *u* ∈ *U*, let *R*(*u*) = {*v* ∈ *N*_*G*_(*u*)∣*u* *v* ∈ *R*} and *B*(*u*) = *N*_*G*_(*u*) ∖ *R*(*u*) and construct a partition *P*(*u*) = {*S*_1_, *S*_2_,…,*S*_*k*(*u*)_}of *N*_*G*_(*u*) as follows.

#### **Partition**

For each *u* ∈ *U*,

(i) put *x*, *y* ∈ *R*(*u*)in the same set of *P*(*u*)if and only if *x* and *y* are in the same connected component of *G* ^′^ = *G* − *E*_*G*[*R*(*u*)]_ − *u*,
(ii) for each *x* ∈ *R*(*u*), put *y* ∈ *B*(*y*)in the same set with *x* if *x* *y* ∈ *E*_*G*_,
(iii) if at least one of the following holds, then return a no-answer and stop:
*P*(*u*)is not a partition of *N*_*G*_(*u*),
  there is a set of *P*(*u*)with at least three vertices of *R*(*u*),
  there is a vertex of *B*(*u*)that is not in a set of *P*(*u*)with a vertex of *R*(*u*),
  there are distinct *S*, *S* ^′^∈ *P*(*u*)such that for some *x* ∈ *S* and *y* ∈ *S* ^′^, *x* *y* ∈ *R*,
  there are distinct *S*, *S* ^′^∈ *P*(*u*)such that for some *x* ∈ *S* and *y* ∈ *S* ^′^, *x* *y* ∈ *E*_*G*_but *u* *x*∉*R* or *u* *y*∉*R*,
  there are distinct *S*, *S* ^′^∈ *P*(*u*)such that for some *x* ∈ *S* and *y* ∈ *S* ^′^, *x* *y*∉*E*_*G*_but *u* *x* ∈ *R* and *u* *y* ∈ *R*,
  the graph *G* − *E*_*G*[*R*(*u*)]_ − *u* has a path connecting vertices of distinct sets of *P*(*u*).

By Lemmas 7, 8 and Claim C, we have the following.

#### **Claim D**

*If* *G* *has a cactus root, then Partition does not stop in Step* *(iii), and if* *H* *is a minimal* *cactus root of* *G*, *then*

(i) *R* ⊆ *E*_*H*_ *and* *B* ∩ *E*_*H*_ = *∅*,
(ii) *every important cut vertex* *u* *of* *H* *is in* *U*,
(iii) *for any* *u* ∈ *U*, *x*, *y* ∈ *N*_*G*_(*u*) *are in the same connected component of* *H* − *u* *if and only if* *x* *and* *y* *are in the same set of* *P*(*u*).

Now we split the instance (*G*, *R*, *B*) of Cactus Root with Labels into several instances of the problem.

#### **Splitting**

For each *u* ∈ *U*, let *P*(*u*) = {*S*_1_,…,*S*_*k*_} and do the following:

(i) delete *u* and introduce *k* new vertices *u*_1_,…,*u*_*k*_,
(ii) for each *i* ∈{1,…,*k*}, make *u*_*i*_adjacent to all vertices of *S*_*i*_,
(iii) for each *i* ∈{1,…,*k*}and *v* ∈ *S*_*i*_, if *u* *v* ∈ *R*, then replace *u* *v* by *u*_*i*_ *v* in *R*, and if *u* *v* ∈ *B*, then replace *u* *v* by *u*_*i*_ *v* in *B*,
(iv) for each *i*, *j* ∈{1,…,*k*}, *i*≠*j*, delete the edges *x* *y* with *x* ∈ *S*_*i*_and *y* ∈ *S*_*j*_,
(v) for each *i* ∈{1,…,*k*}and *v* ∈ *S*_*i*_, update *P*(*v*)by replacing *v* by *v*_*i*_in the sets and deleting the vertices of *N*_*G*_(*u*) ∖ *S*_*i*_from the sets.

Let *G*_1_,…,*G*_*r*_be the connected components of the obtained graph. For *i* ∈{1,…,*r*}, let *R*_*i*_ = *R* ∩ *E*_*G*__*i*_ and *B*_*i*_ = *B* ∩ *E*_*G*__*i*_. By Claims B and D, we establish the following crucial claim.

#### **Claim E**

*The input graph* *G* *has a cactus root if and only if* (*G*_*i*_, *R*_*i*_, *B*_*i*_) *is a yes-instance of* Cactus Root with Labels *for each* *i* ∈{1,…,*r*}. *Moreover, if* (*G*_*i*_, *R*_*i*_, *B*_*i*_) *is a yes-instance, then* *G*_*i*_ *has a cactus root* *H* *with* *R*_*i*_ ⊆ *E*_*H*_ *and* *B*_*i*_ ∩ *E*_*H*_ = *∅* *such that every cut vertex of* *H* *belongs to at most eight blocks and to at most two blocks not being a* *K*_2_.

By Claim E, if *G* has a cactus root, then Δ(*G*_*i*_) ≤ 10 for *i* ∈{1,…,*k*}. By Observations 2 and 3, we obtain that **t** **w**(*G*_*i*_) ≤ 32 in this case. We use Lemma 1 to check whether this holds for each *i* ∈{1,…,*r*}. If the algorithm reports that **t** **w**(*G*_*i*_) ≥ 33 for some *i* ∈{1,…,*r*}, then we return a no-answer and stop. Otherwise, we solve Cactus Root with Labels for each instance (*G*_*i*_, *R*_*i*_, *B*_*i*_) using Lemma 9 for *i* ∈{1,…,*r*}.

It remains to evaluate the running time of our algorithm. We can find all simplicial vertices and sort them into the equivalence classes with the true twin relation in time *O*(*n* ^3^). This implies that the exhaustive application of the Pendant reduction rule can be done in time *O*(*n* ^3^). For each vertex *u* ∈ *V*_*G*_, we can compute the distances between the vertices of *G* − *u* in time *O*(*n* ^3^). Hence, the Labelling step can be done in time *O*(*n* ^4^). For each *u* ∈ *U* the sets *R*(*u*) and *B*(*u*) can be constructed in time *O*(*n* ^2^). For each *u* ∈ *U*, we can construct *G* ^′^ = *G* − *E*_*G*[*R*(*u*)]_ and find the connected components of *G* ^′^in time *O*(*n* ^2^). It follows, that the Partition step can be done in time *O*(*n* ^3^). The Splitting step takes *O*(*n* ^3^) time. The algorithm in Lemma 1 runs in *O*(*n*) time. We conclude that the total running time is *O*(*n* ^4^).

## Squares of Low Clique Number

We first consider the class of 3-degenerate graphs. We will show that 3-degenerate squares have bounded treewidth. In order to do this we need the following two known lemmas.

### **Lemma 10** ([4])

*The* Square Root *problem can be solved in time* *O*(*f*(*t*)*n*) *for* *n* *-vertex* *graphs of treewidth at most* *t*.

### **Lemma 11** ([14])

*Let* *H* *be a square root of a graph* *G*. *Let* *T* *be the bipartite graph with* $V_{T}=\mathcal {C}\cup \mathcal {B}$, *where partition classes* $\mathcal {C}$ *and* $\mathcal {B}$ *are the set of cut vertices and blocks of* *H*, *respectively, such that* $u\in \mathcal {C}$ *and* $Q\in \mathcal {B}$ *are adjacent if and only if* *Q* *contains* *u*. *For* $u\in \mathcal {C}$, *let* *X*_*u*_ *consist of* *u* *and all neighbours of* *u* *in* *H*. *For* $Q\in \mathcal {B}$, *let* *X*_*Q*_ = *V*_*Q*_. *Then* (*T*, *X*) *is a tree decomposition of* *G*.

We call the tree decomposition (*T*, *X*)of Lemma 11 the *H*-*tree decomposition* of *G* and are now ready to prove the following lemma.

### **Lemma 12**

*If* *G* *is a 3-degenerate graph with a square root, then* **t** **w**(*G*) ≤ 3.

### *Proof*

Without loss of generality we assume that *G* is connected and has at least one edge. Let *H* be a square root of *G*. Let $\mathcal {C}$ be the set of cut vertices of *H*, and let $\mathcal {B}$ be the set of blocks of *H*. We construct the *H*-tree decomposition (*T*, *X*)of *G*(cf. Lemma 11). We will show that (*T*, *X*)has width at most 3.

We start with two useful observations. If *v* ∈ *V*_*H*_, then *N*_*H*_[*v*]is a clique in *G*. Because *G* is 3-degenerate, this means that Δ(*H*) ≤ 3. For the same reason *H* contains no cycles of length at least 5 as a subgraph, because a square of a cycle of length at least 5 has minimum degree 4.

We claim that *X*_*Q*_has size at most 4 for every $Q\in \mathcal {B}$. In order to see this, let *Q* be a block of *H*, and let *u* ∈ *V*_*Q*_. Suppose that *Q* has a vertex *v* at distance at least 3 from *u*. Because *Q* is 2-connected, *Q* has two internally vertex disjoint paths that join *u* and *v* and, therefore, *Q*(and thus *H*) contains a cycle of length at least 6 which, as we saw, is not possible. We find that each vertex *v* ∈ *V*_*Q*_is at distance at most 2 from *u*. Hence, *u* is adjacent to all other vertices of *Q* in *G*. By the same reasoning any two vertices in *Q* are of distance at most 2 of each other. Hence, *Q* is a clique in *G*. As *G* is 3-degenerate, this means that *Q* is a clique in *G* of size at most 4. Consequently, *X*_*Q*_, has size at most 4. As Δ(*H*) ≤ 3, we find that *X*_*u*_has size at most 4 for every cut vertex *u* of *H*. □

Lemma 12, combined with Lemmas 1 and 10, leads to the following result.

### **Theorem 2**

Square Root *can be solved in* *O*(*n*) *time for 3-degenerate graphs on* *n* *vertices.*

### *Proof*

Let *G* be an 3-degenerate graph on *n* vertices. By Lemma 1 we can check in *O*(*n*)time whether **t** **w**(*G*) ≤ 3. If **t** **w**(*G*) > 3, then *G* has no square root by Lemma 12. If not we solve Square Root in *O*(*n*)time by using Lemma 10. □

### *Remark 1*

**Fig. 3:**
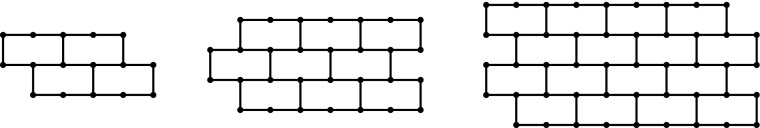
Walls of height 2, 3, and 4, respectively

We cannot claim any upper bound for the treewidth of 4-degenerate graphs with a square root. In order to see this, take a wall (see Fig. 3) and subdivide each edge three times, that is, replace each edge *u* *v* by a path *u* *a* *b* *c* *v* where *a*, *b*, *c* are three new vertices. This gives us a graph *H*, such that *H* ^2^is 4-degenerate. In order to see the latter, note that every “*b*-type” vertex has degree 4 in *H* ^2^and that after removing all degree-4 vertices, we obtain a disjoint number of copies of *K*_4_, each of which is 4-degenerate. A wall of height *h* has treewidth Ω(*h*)(see, for example, [8]). As subdividing an edge and adding edges does not decrease the treewidth of a graph, this means that the graph *H* ^2^can have arbitrarily large treewidth.

We now consider (*K*_*r*_, *P*_*t*_)-free graphs (that is, graphs with no induced path *P*_*t*_ and no complete subgraph *K*_*r*_). We let *K*_*s*, *s*_denote the complete bipartite graph in which both partition classes have *s* vertices. We need a result of Atminas, Lozin and Razgon.

### **Lemma 13** ([2])

*For any two integers* *s* *and* *t*, *there exists an integer* *b*(*s*, *t*) *such that any graph of treewidth at least* *b*(*s*, *t*) *contains the path* *P*_*t*_ *as an induced subgraph or the complete bipartite graph* *K*_*s*, *s*_ *as a (not necessarily induced) subgraph.*

Lemma 13, together with Ramsey’s Theorem, enables us to prove the following lemma.

### **Lemma 14**

*For every two integers* *r*, *t* ≥ 1, *the class of* (*K*_*r*_, *P*_*t*_) *-free* *graphs with a square root has bounded treewidth.*

### *Proof*

Let *r*, *t* ≥ 1. For contradiction, assume that the class of (*K*_*r*_, *P*_*t*_)-free graphs with a square root has unbounded treewidth. Then there exists a (*K*_*r*_, *P*_*t*_)-free graph *G* with a square root such that *G* has treewidth at least *b*(*s*, *t*), where *b*(*s*, *t*)is the constant in Lemma 13 for a sufficiently large integer *s*. Then, by Lemma 13, we find that *G* contains a subgraph *F* isomorphic to *K*_*s*, *s*_. As we have chosen the fixed integer *s* to be large enough, Ramsey’s Theorem implies, together with the *K*_*r*_-freeness of *G*, that *F* is in fact an induced subgraph of *G*. This means that no two vertices in the same bipartition class of *F* may have a common neighbour in *H*. In particular, this implies that for each *u* ∈ *V*_*F*_, *H* contains at most one edge of *F* incident to *u*(as otherwise *u* would be a common neighbour of two vertices of *F* in *H*).

Let *A* and *B* be the bipartition classes of *F*. Let *u* ∈ *A*. As *s* is sufficiently large, there exist at least *r* vertices *v*_1_,…,*v*_*r*_in *B* that are not adjacent to *u* in *H*. Then there must exist *r* distinct vertices *w*_1_,…,*w*_*r*_with edges *u* *w*_*i*_and *w*_*i*_ *v*_*i*_for *i* = 1,…,*r*(to enforce the edges *u* *v*_*i*_in *G* for *i* = 1,…,*r*). As the vertices *w*_1_,…,*w*_*q*_all have common neighbour *u* in *H*, they form a clique of size *r* in *G*, a contradiction with the *K*_*r*_-freeness of *G*. □

Using a similar reasoning as before, we find that Lemma 14, combined with Lemmas 1 and 10, leads to the following result.

### **Theorem 3**

*For every two integers* *r*, *t* ≥ 1, Square Root *can be solved in time* *O*(*n*) *for* (*K*_*r*_, *P*_*t*_) *-free* *graphs on* *n* *vertices.*

## Conclusions

We proved that the problem of testing whether a graph has a cactus root is *O*(*n* ^4^)-time solvable. In fact, our algorithm can be modified to find a cactus root in the same time (if it exists). Every cactus is outerplanar, and recently Golovach et al. [12] proved that squares of outerplanar graphs can be recognized in polynomial time. Determining the complexity of $\mathcal {H}$-Square Root when $\mathcal {H}$ is the class of planar graphs is still a wide open problem. Golovach et al. [12] also proved that squares of graphs of pathwidth at most 2 can be recognized in polynomial time. We recall that every cactus has treewidth at most 2. This leads to the open problem of determining the complexity of $\mathcal {H}$-Square Root when $\mathcal {H}$ is the class of graphs of treewidth at most 2. We also recall that a cactus is a connected graph, in which each block is either a cycle or an edge. This leads to the following (known) generalization: a *cactus block graph* is a connected graph, in which each block is a cycle or a complete graph. Recently, Ducoffe [9] gave a polynomial-time algorithm for the problem of recognizing squares of cactus block graphs. In Table 1 we summarize all known results on $\mathcal {H}$-Square Root.

**Table 1:** A survey of the known results for $\mathcal {H}$-Square Root (the result marked with a ∗is proven in this paper)

Graph class $\mathcal {H}$	complexity
Trees [23]	polynomial
Proper interval graphs [18]	polynomial
Bipartite graphs [17]	polynomial
Block graphs [21]	polynomial
Strongly chordal split graphs [22]	polynomial
Ptolemaic graphs [19]	polynomial
3-sun-free split graphs [19]	polynomial
Cactus graphs^∗^	polynomial
Cactus block graphs [9]	polynomial
Graphs of pathwidth at most 2 [12]	polynomial
Outerplanar graphs [12]	polynomial
Graphs with girth at least *g* for any fixed *g* ≥ 6 [11]	polynomial
Graphs of girth at least 5 [10]	NP-complete
Graphs of girth at least 4 [11]	NP-complete
Split graphs [18]	NP-complete
Chordal graphs [18]	NP-complete

The result for 3-sun-free split graphs has been extended to a number of other subclasses of split graphs in [20]

We observed that Square Root is polynomial-time solvable for graphs with clique number 3 (or equivalently, *K*_4_-free graphs) and proved the same result for 3-degenerate graphs and (*K*_*r*_, *P*_*t*_)-free graphs for every *r*, *t* ≥ 1. We summarize the known results for Square Root in Table 2. As can be seen from this table, the computational complexity of Square Root is unknown for several well-known graph classes. In particular, we recall the open problems of Milanič and Schaudt [25], who asked about the complexity of Square Root restricted to split graphs and cographs. We also do not know the computational complexity of Square Root for *K*_*r*_-free graphs for *r* ≥ 5 and for graphs of maximum degree at most *s* for *s* ≥ 7.

**Table 2:** A survey of the known results for Square Root restricted to some special graph class $\mathcal {G}$

Graph class $\mathcal {G}$	complexity
Planar graphs [23]	linear
Non-trivial and minor-closed [28]	linear
*K*_4_-free graphs^∗^	linear
(*K*_*r*_, *P*_*t*_)-free graphs^∗^	linear
3-degenerate graphs^∗^	linear
Graphs of maximum degree ≤ 5 [4]	linear
Graphs of maximum degree ≤ 6 [4]	polynomial
Graphs of maximum average degree $<\frac {46}{11}$ [14]	polynomial
Line graphs [24]	polynomial
Trivially perfect graphs [25]	polynomial
Threshold graphs [25]	polynomial
Chordal graphs [18]	NP-complete

Note that the row for planar graphs is absorbed by the row directly below it. Results marked with a ∗are results shown in this paper
